# Prokaryotic Communities in the Thalassohaline Tuz Lake, Deep Zone, and Kayacik, Kaldirim and Yavsan Salterns (Turkey) Assessed by 16S rRNA Amplicon Sequencing

**DOI:** 10.3390/microorganisms9071525

**Published:** 2021-07-17

**Authors:** Can Akpolat, Ana Beatriz Fernández, Pinar Caglayan, Baris Calli, Meral Birbir, Antonio Ventosa

**Affiliations:** 1 Department of Biology, Faculty of Arts and Sciences, Marmara University, 34722 Istanbul, Turkey; canakpolat@marun.edu.tr (C.A.); pinar.caglayan@marmara.edu.tr (P.C.); 2 Institute for Multidisciplinary Research in Applied Biology-IMAB, Universidad Publica de Navarra, Multilva, 31006 Navarra, Spain; anabeatriz.fernandez@unavarra.es; 3 Department of Environmental Engineering, Faculty of Engineering, Marmara University, 34722 Istanbul, Turkey; baris.calli@marmara.edu.tr; 4 Department of Microbiology and Parasitology, Faculty of Pharmacy, University of Sevilla, 41004 Sevilla, Spain

**Keywords:** Tuz Lake (Salt Lake), thalassohaline lakes, salterns, metagenomics, extremophiles, 16S rRNA amplicon sequencing, physico-chemical analyses

## Abstract

Prokaryotic communities and physico-chemical characteristics of 30 brine samples from the thalassohaline Tuz Lake (Salt Lake), Deep Zone, Kayacik, Kaldirim, and Yavsan salterns (Turkey) were analyzed using 16S rRNA amplicon sequencing and standard methods, respectively. *Archaea* (98.41% of reads) was found to dominate in these habitats in contrast to the domain *Bacteria* (1.38% of reads). Representatives of the phylum *Euryarchaeota* were detected as the most predominant, while 59.48% and 1.32% of reads, respectively, were assigned to 18 archaeal genera, 19 bacterial genera, 10 archaeal genera, and one bacterial genus that were determined to be present, with more than 1% sequences in the samples. They were the archaeal genera *Haloquadratum*, *Haloarcula*, *Halorhabdus*, *Natronomonas*, *Halosimplex*, *Halomicrobium*, *Halorubrum*, *Halonotius*, *Halolamina*, *Halobacterium*, and *Salinibacter* within the domain *Bacteria*. The genera *Haloquadratum* and *Halorhabdus* were found in all sampling sites. While *Haloquadratum*, *Haloarcula*, and *Halorhabdus* were the most abundant genera, two uncultured Tuz Lake *Halobacteria* (TLHs) 1 and 2 were detected in high abundance, and an additional uncultured haloarchaeal TLH-3 was found as a minor abundant uncultured taxon. Their future isolation in pure culture would permit us to expand our knowledge on hypersaline thalassohaline habitats, as well as their ecological role and biomedical and biotechnological potential applications.

## 1. Introduction

Hypersaline habitats such as salt lakes, saline soils, solar salterns, hypersaline soda lakes, salt mines, and deep-sea and oil reservoir brines are typical extreme environments in which not only NaCl, but other environmental factors, such as pH, temperature, nutrients, radiation, pressure or presence of heavy metals and other toxic compounds, limit their microbiota [1,2,3,4,5,6,7,8].

Hypersaline aquatic environments are classified as thalassohaline habitats; those derived from marine origin having relative proportions of salts according to those of seawater, and athalassohaline aquatic systems, with salts proportions very different from seawater, reflecting their non-marine and very different geological origins [1,2,4]. Most studies on hypersaline lakes have been focused on athalassohaline lakes, such as the Dead Sea, hypersaline and alkaline (soda) lakes in Antarctica, East African lakes such as Lake Magadi and the lakes of Wadi Natrun, in Australia or China, especially in Inner Mongolia region, and deep-sea lakes [1,2,8,9,10,11,12,13,14]. These environments, which host a wide variety of microorganisms belonging to *Archaea* and *Bacteria*, have attracted considerable attention in recent years in terms of the microbial diversity and their adaptations to high salt concentrations, as well as on the biotechnological and industrial applications of these extremophilic microorganisms [5,15,16,17]. However, relatively little is known about the prokaryotic diversity in thalassohaline lakes, including the hypersaline Tuz Lake and its associated salterns, in Turkey.

**Figure 1:**
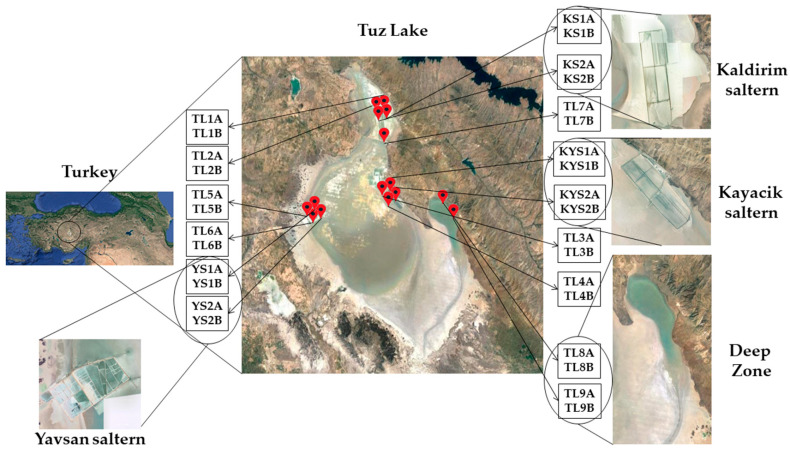
Locations of the sampling sites and 30 brine samples collected from Tuz Lake, Deep Zone, and Kayacik, Kaldirim, and Yavsan salterns in Turkey.

The hypersaline Tuz Lake (in Turkish Tuz Gölü), located in the Konya Closed Basin on the Central Anatolian Peninsula, is the largest hypersaline lake in Turkey, with a maximum surface area of 1665 km^2^ [18]. It has an average altitude of 905 m and is surrounded by the Kızılırmak massif in the east, Obruk Plateau in the south, Cihanbeyli Plateau in the west, and the Haymana Plateau in the north [19]. Tuz Lake constitutes the Main Zone (a water body with a depth of about 70 cm in the spring) and a separate area of the lake, designated as the Deep Zone (a water body with a depth exceeding 100 cm in the spring), is found in the mid-east part of the main area of the lake [20,21]. In addition, on the shore of Tuz Lake, there are several salterns that are the major source for salt production in Turkey, estimated to meet 70% of the salt needs of this country [22,23]. Kayacik saltern, Kaldirim saltern, and Yavsan saltern are located in the mid-east, north-east, and western parts of the lake, respectively (Figure 1). The lake is a closed water basin where the water partly filters into the soil and partly evaporates. The lake is mostly fed by underground water and partially by the small rivers that come from Konya Closed Basin and Obruk Plateau. In summer, most of the incoming streams dry up and do not reach the lake. Due to the excessive evaporation, the lake dries out almost completely and about 30 cm thick salt layers are formed. The average salt content and density of the lake water are 32.4% and 2–2.5 g/cm^3^, respectively [24]. The annual average temperature is 12 °C (1.6 °C in winter and 22.2 °C in summer, respectively), and the mean annual precipitation is 324 mm/m^2^. Tuz Lake and the surrounding area have been determined as the most arid regions in Turkey.

Some pollution load reaches the Tuz Lake from various sources located around the lake, especially sulfate-rich wastewater from a sodium sulfate manufacturing plant in the southwest, and partially treated domestic wastewater discharged into the small creeks feeding the lake, and to a lesser extent the metal residue particles of the mining activities and fertilizer and pesticide residues originating from the agricultural activities in the region [19].

The prokaryotic diversity of Tuz Lake has been mostly investigated using culture-dependent methods, permitting the isolation of moderately halophilic bacterial and extremely halophilic archaeal strains that produced lipases and proteases, and to a lower extent cellulase and β-galactosidase enzymes [22,25,26,27,28]. More recently, the archaeal and bacterial diversity of the lake, using culture-independent methods, has been investigated [18,29,30,31,32]. A higher percentage of the archaeal community than that of the bacterial community was detected by FISH. Based on the 16S rRNA gene library data, *Haloquadratum* and *Salinibacter ruber* related phylotypes were found as the predominant microorganisms [29]. In another culture-independent study carried out in Tuz Lake, in the five brine samples studied, 29 archaeal and 23 bacterial OTUs were detected using 16S rDNA metabarcoding. The OTUs of *Haloquadratum walsbyi* and the genus *Salinibacter* were found as the most predominant in these samples [30]. Moreover, the bacterial composition of Tuz Lake was examined by the V3–V4 variable regions of bacterial 16S rRNA gene. *Firmicutes*, *Fusobacteria*, and *Proteobacteria* were determined as the most abundant bacterial phyla in two brine samples of the lake [31].

In a recent study, the bacterial and archaeal diversity found in 11 sediment samples collected from Cihanbeyli and Şereflikoçhisar regions of Tuz Lake were examined by the comparison of the V4 region of 16S rRNA gene, using Illumina MiSeq sequencing platform and cloning. While *Gammaproteobacteria*, *Bipolaricaulia*, *Desulfovibrionia*, candidate division MSBL-1, *Bacteroidia*, and *Desulfobacteria* were detected as the most abundant classes, *Gemmatimonadota*_c, *Rhodothermia*, *Alphaproteobacteria*, *Methanonatronarchaeia*, *Thermoplasmata*, *Halanaerobiia*, *Desulfobulbia*, and *Bacilli* were found as low abundance classes [18].

Overall, previous microbiological studies using culture-independent analyses were limited to a small number of samples and sampling areas compared to the great extent of the lake or were restricted to the study of bacteria or sediments of Tuz Lake [18,29,30,31]. In this study, we have determined the composition and abundance of prokaryotic communities present in the brines of different sampling sites of Tuz Lake, including the Deep Zone, as well as the three salterns, Kayacik, Kaldirim, and Yavsan that are associated with the lake, through 16S rRNA gene amplicon sequencing.

## 2. Materials and Methods

### 2.1. Brine Sample Collection

Thirty brine samples were collected in July 2017 from 15 different sampling sites (2 brine samples per site), as shown in Figure 1. The Deep Zone, with a depth of about 1 m, is located at the east of Tuz Lake and was previously part of the lake. However, drought has caused a permanent separation of this area from Tuz Lake. Kayacik, Kaldirim, and Yavsan salterns are located on the shores of the Tuz Lake.

The samples were coded according to the sampling sites: Tuz Lake (TL1A–TL1B, TL2A–TL2B, TL3A–TL3B, TL4A–TL4B, TL5A–TL5B, TL6A–TL6B, TL7A–TL7B), Deep Zone (TL8A–TL8B, TL9A–TL9B), Kayacik saltern (KYS1A–KYS1B, KYS2A–KYS2B), Kaldirim saltern (KS1A–KS1B, KS2A–KS2B), and Yavsan saltern (YS1A–YS1B, YS2A–YS2B). The brine samples were collected in sterile bottles, carried to the laboratory on ice in the dark, and processed immediately (Figure 1).

### 2.2. Physico-Chemical Analyses

In-situ measurements of temperature and pH of the brine samples were performed using a portable pH meter (PT 10, Sartorius Professional Meter PP–50 AG, Göttingen, Germany). The salinity of the brine samples was measured using a hand refractometer (Reef Octopus, China). Chloride concentrations of the brines were determined by argentometric method [33,34]. Sodium, potassium, magnesium, and calcium concentrations in the brine samples were determined using a flame atomic absorption spectroscopy (FAAS) (Perkin Elmer, AAS 400, Waltham, MA, USA) via air-acetylene flame [35]. Linear ranges used in the analyses were 10–100 mg L^−1^ for Na^+^, 1–20 mg L^−1^ for K^+^, 0.1–2 mg L^−1^ for Mg^2+^, and 20–100 mg L^−1^ for Ca^2+^.

### 2.3. DNA Extraction from the Brine Samples

A total of 1.5 L of the brine sample was sequentially filtered through 5 µm and 0.2 µm pore size filters, using a peristaltic pump [36,37,38]. Cells obtained from the filters were resuspended in 2.5 M NaCl, then separately placed into sterile tubes and centrifuged at 13,000 rpm for 30 min. The environmental DNA was extracted from each sample using the PowerSoil DNA isolation kit (Qiagen, USA), according to the manufacturer’s protocol [38]. The quantity and quality of the extracted DNA were analyzed by a Nanodrop spectrophotometer (NanoDrop 2000) at 260/280 nm and 260/230 nm, and by agarose gel electrophoresis. The extracted DNA samples were stored at −20 °C until further processing [39].

### 2.4. 16S rRNA Amplicon Processing from the Extracted DNA Samples

The V6–V8 variable regions of archaeal and bacterial 16S rRNA genes of the extracted DNA belonging to the brine samples were amplified using forward primer 926F (5′-AAACTYAAAKGAATTGRCGG-3′) and reverse primer 1392R (5′-ACGGGCGGTGTGTRC-3′) [40]. PCR amplification was performed using the HotStarTaq Plus Master Mix (Qiagen, USA). The following PCR conditions were used: initial denaturation at 94 °C for 3 min, followed by 28 cycles at 94 °C for 30 s, at 53 °C for 40 s, and 72 °C for 60 s, followed by final extension step at 72 °C for 5 min. After the PCR amplification step, PCR products were run on 2% agarose gel. The concentration of each PCR product was measured and pooled equimolarly. The pooled amplicons were purified using AMPure XP (Beckman Coulter, Brea, CA, USA) magnetic beads. The pooled and purified amplicons were subjected to NGS library preparation protocol for Illumina. Sequencing was performed on the Illumina MiSeq platform with 300 bp paired-end chemistry (Macrogen Inc., Seoul, Korea).

### 2.5. Bioinformatic Analysis

The quality of the raw data was evaluated using the FastQC program [41]. 16S rRNA gene amplicon data were merged and analyzed with the QIIME program [42]. Demultiplexing was performed using barcode sequences specific to each sample. Then, the Illumina MiSeq reads were quality filtered by removing low-quality or ambiguous reads. All barcode and primer sequences were removed and the reads were clustered at 97% identity in operational taxonomic units (OTUs) using UCLUST [43]. A representative sequence for each OTU was selected and all representative OTU sequences were aligned with PyNAST v1.2.2 [44] using the Greengenes (GreenGenes v13.8) database. Chimeric fragments were detected with the ChimeraSlayer algorithm [45] and all chimeric reads were excluded. In addition, OTUs with only one read were discarded. Then, the OTU table was formed, including information of read counts of each OTU belonging to the sample. Taxonomic classification of the OTU table was performed using the UCLUST program and Greengenes database. Alpha diversity analyses were also performed with the QIIME program using the OTU table and diversity indexes (PD Whole Tree, Chao1, Observed OTUs, Shannon, Simpson, and Dominance) were calculated. Beta diversity analyses were performed using weighted and unweighted unifrac metrics and visualized as principal component analysis (PCA) graphs. Rarefaction curves were generated by QIIME pipeline v1.9.1 [42].

### 2.6. Statistical Analyses

In the present study, statistical correlations among the abundance of archaeal and bacterial genera were performed using the R software. The correlation among different variables was analyzed by the “cor” function using the R software. Principal component analysis was made by the “prcomp” function [46].

### 2.7. Sequencing Data

All sequence data obtained in this study are freely available at the European Nucleotide Archive (ENA)/NCBI under the accession number PRJNA705280.

## 3. Results

### 3.1. Physico-Chemical Analyses

The 30 brine samples collected from Tuz Lake, Deep Zone, and Kayacik, Kaldirim, and Yavsan salterns were analyzed to determine the physico-chemical characteristics (Table S1). High salinity and near-neutral pH values were observed in all brine samples. Salinity of the samples from Tuz Lake and Deep Zone ranged from 30 to 38%, with intermediate values of 32, 34, and 36% from several sampling sites. The salinity of the samples from Kayacik, Kaldirim, and Yavsan salterns was also very high, with percentages of 30–32, 32–36, and 32%, respectively. The pH of the samples ranged between 6.9 (only two sampling sites) and 7.4, and the temperature of the brine samples was very constant, ranging from 25 to 27 °C, except for some of Tuz Lake samples and all Deep Zone samples for which the temperature was 22 and 24 °C. Na^+^ and Cl^−^ contents were fairly high in all brine samples. Although K^+^ and Mg^2+^ contents were found as moderate, low Ca^2+^ concentrations were determined for all samples studied (Table S1). Overall, these data, especially the neutral pH and high content of Na^+^ and Cl^−^ concentrations, in contrast to divalent cations, corroborate the thalassohaline character of Tuz Lake and the salterns investigated in this study.

### 3.2. Alpha Diversity and Richness Metrics

In the present study, a total of 1,282,558 16S rRNA gene sequences obtained from the 30 brine samples were clustered into 17,488 OTUs at 97% sequence similarity. YS2A sample, obtained from Yavsan saltern, had the maximal values according to both, the number of sequences (97,361) and OTUs (5725). In contrast, the Tuz Lake and Deep Zone samples TL4A and TL8B had minimal values in terms of the sequence count (26,179) and OTU number (707), respectively.

Although samples from Tuz Lake and Deep Zone showed slopes on the rarefaction curves close to zero or gentle slope, samples from Kayacik, Kaldirim, and Yavsan salterns had raised slopes, suggesting that the deep sequencing was insufficient to cover the diversity in these few samples (Figure S1).

To examine the diversity and richness in Tuz Lake, Deep Zone, and Kayacik, Kaldirim, and Yavsan salterns, observed OTUs, PD whole tree, Chao1, Shannon, Simpson, and Dominance indexes were calculated. Chao1 and Shannon indexes indicated that the highest diversities were detected in TL2 and KS1 sampling regions and the lowest diversities were detected in TL8–TL9 sampling sites corresponding to the Deep Zone (Table 1). Brine samples of the Deep Zone had lower OTU numbers than those of brine samples of Tuz Lake and its salterns, and consequently, lower prokaryotic diversity was detected at this region because of a large dominance of a few OTUs (Table 1).

### 3.3. Prokaryotic Communities in Tuz Lake, Deep Zone, and Kayacik, Kaldirim, and Yavsan Salterns

Our analyses indicated that most 16S rRNA gene sequences were classified within the domain *Archaea* (98.41%) and clustered into 16,939 OTUs. In contrast, only a few sequences could be assigned to the domain *Bacteria* (1.38%) and they clustered into 218 OTUs. Fifty-one OTUs belonging to the archaeal class *Halobacteria* and one OTU belonging to the bacterial class *Rhodothermia*, family *Salinibacteraceae* were determined in all the brine samples collected from Tuz Lake, Deep Zone, and Kayacik, Kaldirim, and Yavsan salterns.

**Figure 2:**
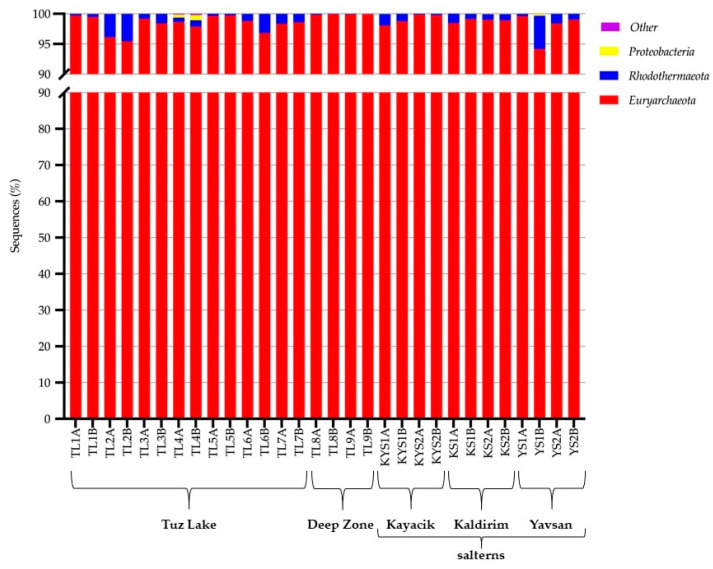
Phyla distributions of prokaryotic communities determined in brine samples of Tuz Lake (TL1A to TL7B), Deep Zone (TL8A to TL9B), and Kayacik (KYS1A to KYS2B), Kaldirim (KS1A to KS2B), and Yavsan (YS1A to YS2B) salterns.

The archaeal phylum *Euryarchaeota* (93.76–99.94%, with a mean of 98.41 ± 0.27%), and the bacterial phyla *Rhodothermaeota* (0.02–5.52%, 1.28 ± 0.25%), *Proteobacteria* (0.00–0.83%, 0.06 ± 0.03%), *Firmicutes* (0.00–0.33%, 0.02 ± 0.01%), and *Lentisphaerae* (0.00–0.11%, 0.009 ± 0.005%) were found in the samples. In addition, the archaeal phylum *Parvarchaeota* (0.00–0.003%) and the bacterial phyla *Actinobacteria* (0.00–0.008%, 0.001 ± 0.0003%), *Spirochaetes* (0.00–0.004%) and *Cyanobacteria* (0.00–0.01%, 0.001 ± 0.0005%) were detected at extremely low abundance in a few brine samples. According to these data, *Euryarchaeota*, distantly followed by *Rhodothermaeota*, were the most abundant phyla determined in all brine samples studied (Figure 2).

The phyla composition detected in all brine samples of Tuz Lake, and Kayacik, Kaldirim and Yavsan salterns were fairly similar, except for the Deep Zone (Table S2). It is noteworthy that the two brine replicates collected per site shared a quite similar phyla composition (Figure 2). The highest percentage of *Euryarchaeota* was detected at the brine samples of the Deep Zone. *Rhodothermaeota* was found slightly more abundant in some locations of Tuz Lake (TL2, TL3, TL4, TL6, and TL7 sampling sites), Kayacik saltern (KYS1 samples), Kaldirim saltern (KS1 and KS2 samples), and Yavsan saltern (YS1 and YS2 samples) (Figure 2).

**Figure 3:**
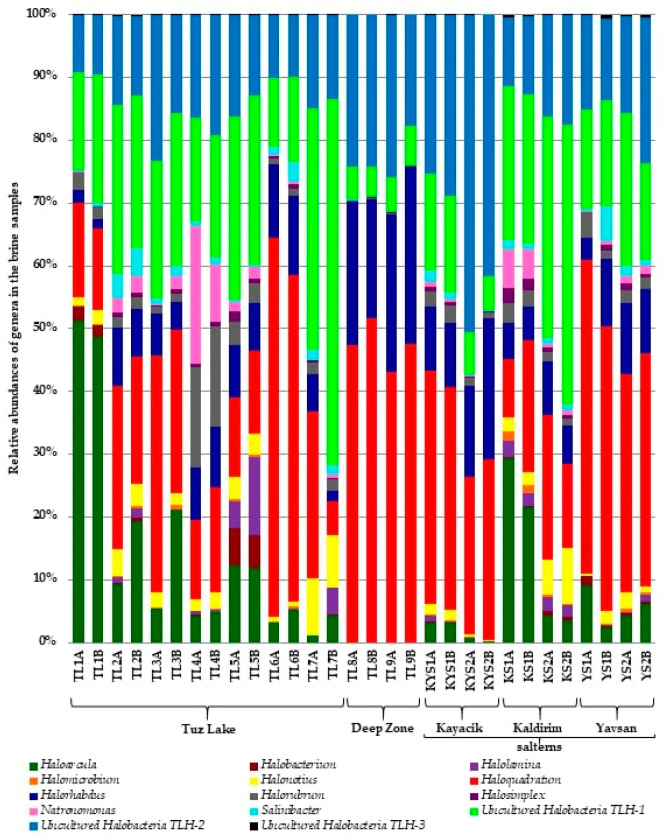
Taxonomic profiles at the genus level of prokaryotic communities detected in brine samples of Tuz Lake (TL1A to TL7B), Deep Zone (TL8A to TL9B), and Kayacik (KYS1A to KYS2B), Kaldirim (KS1A to KS2B), and Yavsan (YS1A to YS2B) salterns. Each OTU contains at least 1% 16S rRNA sequence.

At genus level, most of the sequences were classified in the phylum *Euryarchaeota*, being the OTU classified in the genus *Haloquadratum* remarkably abundant in most of the samples (Figure 3). However, a large number of sequences clustered into OTUs that could not be assigned to any known genera (39% of sequences). Thus, only 61% of sequences obtained in this study were assigned or closely related to previously described genera. Two uncultured OTUs were well represented in these samples and both belonged or were related to the class *Halobacteria*. Besides, the genera *Haloarcula* and *Halorhabdus* also recruited many sequences in these environments. In contrast, only 1.38% of sequences were assigned to *Bacteria* and most of them were related to the genus *Salinibacter* (Table 2 and Figure 3).

*Haloquadratum* (23.99%) and *Haloarcula* (14.36%) were determined as the most predominant genera in the brine samples of Tuz Lake, with the uncultured *Halobacteria* TLH-1 (24.61%) as the most abundant taxon, and lower percentages for the representatives of the uncultured *Halobacteria* TLH-2 (13.98%) and the archaeal genera *Halorhabdus* (6.90%), *Halorubrum* (3.87%), *Halonotius* (3.25%), *Natronomonas* (3.00%), *Halolamina* (1.73%), and *Halobacterium* (1.19%), and the bacterial genus *Salinibacter* (1.54%) among those genera represented by at least 1% of the sequences (Table 2).

The Deep Zone samples were highlighted by a large presence of *Haloquadratum* (47.32%), followed by *Halorhabdus* (23.76%) and the uncultured *Halobacteria* TLH-2 (22.99%). This last scenario is also repeated in Kayacik saltern samples, but in this case, the uncultured *Halobacteria* TLH-2 was more remarkable in these samples (36.18%) than in the others. A similar composition was found in Yavsan saltern samples, but also uncultured *Halobacteria* TLH-1 showed a great abundance (17.93%). In Kaldirim saltern, the most abundant taxon was the uncultured *Halobacteria* TLH-1, followed by *Haloquadratum* and the uncultured *Halobacteria* TLH-2. Finally, in contrast to Deep Zone and the other salterns, the genus *Haloarcula* was well represented in KS1 samples from Kaldirim saltern (Table 2 and Figure 3).

In summary, concerning the domain *Archaea*, the genus *Haloquadratum* was always present and represented the most abundant taxon on most sampling sites (with percentages ranging from 47.32 to 16.57%), while on the domain *Bacteria*, the genus *Salinibacter* was the only representative present at relatively low proportions (Table 2). Finally, two unknown and not yet isolated taxa, designated as uncultured *Halobacteria* TLH-1 and TLH-2, were predominant on Tuz Lake and Kaldirim saltern, and on Kayacik saltern, respectively (Table 2).

### 3.4. Principal Components Analysis (PCA)

**Figure 4:**
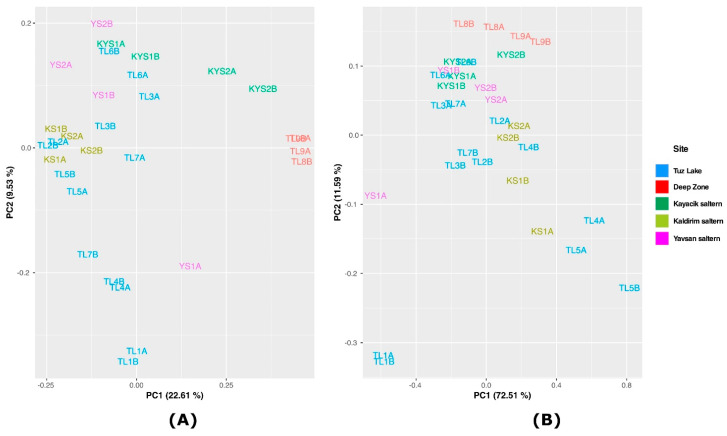
Principal components analysis (PCA) of the brine samples of Tuz Lake, Deep Zone, and Kayacik, Kaldirim, and Yavsan salterns. (**A**) Unweighted UniFrac, (**B**) Weighted UniFrac.

Unweighted and weighted UniFrac, which are measurements of β-diversity, compare prokaryotic communities based on the phylogenetic relationships of the *Archaea* and *Bacteria* contained within communities. The PCA plot provides an overview visualization of the relationships of the communities among the samples [47,48,49]. UniFrac distances were generated using weighted and unweighted methods to compare the detected archaeal and bacterial communities of the brine samples collected from Tuz Lake, Deep Zone, and Kayacik, Kaldirim, and Yavsan salterns in terms of diversity among the samples and plotted them on PCA plots (Figure 4).

Figure 4 shows that the samples from each site were grouped relatively close to each other, except the sample YS1A with a more limited taxonomic diversity than the other samples from Yavsan saltern and the samples from Tuz Lake that were taken from different distant places due to the great extension of this lake. Especially, TL1 brine samples showed a greater difference in their variance compared to the rest of the brine samples collected from Tuz Lake.

A higher variance (84.1%) was obtained from the weighted UniFrac PCA than the unweighted UniFrac PCA using the first two principal components. In this plot, five brine samples of Tuz Lake (TL1A, TL1B, TL4A, TL5A, and TL5B), and one brine sample of Yavsan (YS1A) saltern were not grouped closely with the other brine samples (Figure 4B).

### 3.5. Correlations among Genera

**Figure 5:**
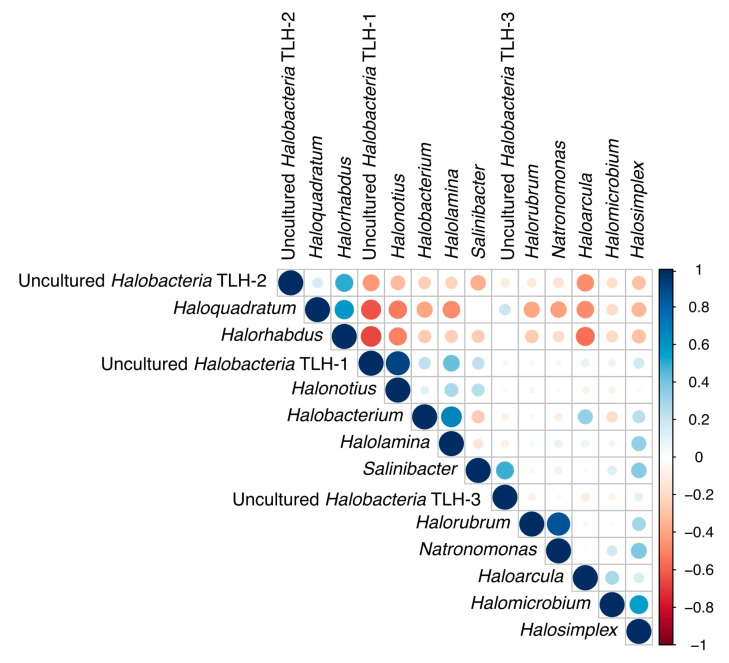
Statistical correlations among the genera with more than 1% sequences detected in Tuz Lake, Deep Zone, and Kayacik, Kaldirim, and Yavsan salterns.

The genera (>1% sequences) detected in the brine samples collected from Tuz Lake, Deep Zone, and Kayacik, Kaldirim, and Yavsan salterns and their statistical correlations were generated using R software version 3.6.2 (Figure 5). Diversity and abundance of the microbial communities changed according to positive and negative correlations among archaeal genera found in each sampling site (*p* < 0.05). Positive strong correlations were found between *Halonotius* and the uncultured *Halobacteria* TLH-1 (r = 0.917); genera *Halorubrum* and *Natronomonas* (r = 0.857); and genera *Halolamina* and *Halobacterium* (r = 0.676). Moreover, the genus *Haloquadratum* was found positively correlated with the genus *Halorhabdus* (r = 0.582) and the uncultured *Halobacteria* TLH-2 (r = 0.159). However, the increased presence of *Haloquadratum*, *Halorhabdus*, and the uncultured *Halobacteria* TLH-2 were negatively correlated with the abundance of the genera *Haloarcula*, *Halobacterium*, *Halolamina*, *Halomicrobium*, *Halonotius*, *Halorubrum*, *Halosimplex, Natronomonas* and the uncultured *Halobacteria* TLH-1. Although these genera had negative correlations with *Haloquadratum*, *Halorhabdus* and the uncultured *Halobacteria* TLH-2, in general, they had positive correlations with each other.

### 3.6. Correlations among Physico-Chemical Parameters and Brine Samples

**Figure 6:**
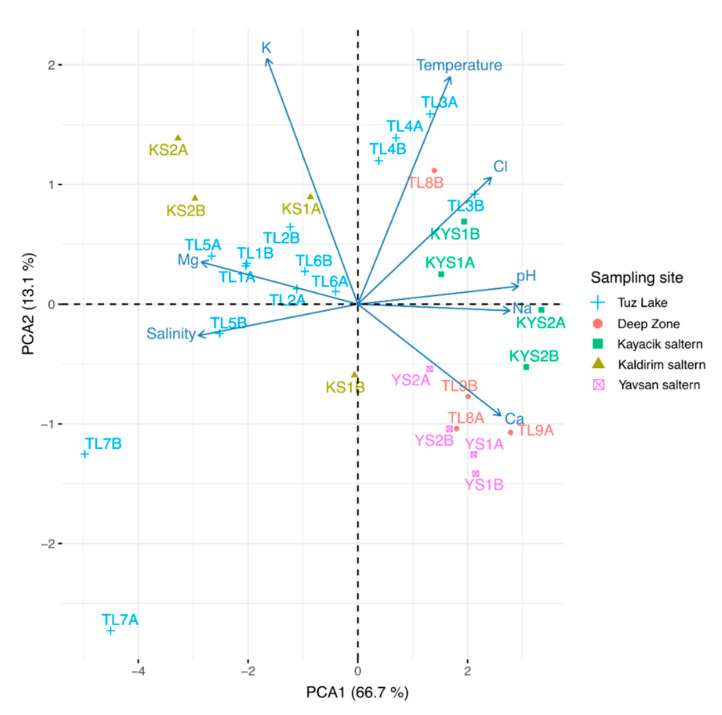
PCA biplot of Tuz Lake, Deep Zone, and Kayacik, Kaldirim, and Yavsan salterns’ brine samples and physico-chemical parameters (blue arrows). The two PCA axes (Dim1 and Dim2) explained 79.8% of data variance. The brine samples are shown in the PCA biplot according to their relationship with the physico-chemical parameters.

According to PCA analysis, eight physico-chemical variables (temperature, pH, salinity, Na^+^, K^+^, Mg^2+^, Ca^2+^, and Cl^−^ concentrations) were reduced to two principal components (Figure 6 and Table S3). Two principal components (Dim1 and Dim2) explained 79.8% of the total variance. While the first component (Dim1) explained most of the variance (66.7%), the second component (Dim2) explained 13.1% of the variance. While pH, salinity, and sodium concentration were determined as the strongest parameters in the first component (Dim1), potassium concentration and temperature were in the second component (Dim2) (Table S3).

As seen in Figure 6, samples of the Deep Zone, Kayacik (KYS2A-KYS2B), and Yavsan salterns were positively correlated to Na^+^, Ca^2+^, and Cl^−^ concentrations, pH, and temperature. Samples from Kaldirim saltern had a higher correlation to the increase of K^+^ and Mg^2+^ concentrations and salinity.

However, TL7A and TL7B brine samples from Tuz Lake were especially located separately due to their different physico-chemical characteristics (Figure 6). Although this sampling site had high salinity and Mg^2+^ concentration, the temperature, pH, Na^+^, Ca^2+^ and Cl^−^ concentrations of this site were found to be low (Table S1). TL3A and TL4 brine samples from Tuz Lake had a strong positive correlation with temperature and Cl^−^ concentration, while TL1, TL2, and TL5 brine samples from Tuz Lake exhibited a positive correlation with salinity and K^+^ and Mg^2+^ concentrations (Figure 6).

## 4. Discussion

The current study presents the physico-chemical analyses, including the ionic composition as well as the diversity and abundance of archaeal and bacterial communities in seven different areas of Tuz Lake and two different locations of the Deep Zone, and the three salterns located on the shore of the lake, namely Kayacik, Kaldirim, and Yavsan salterns in summer season, using 16S rRNA gene based amplicon sequencing on Illumina platform. Furthermore, this study also offers the correlations among the genera and chemical analyses.

Thalassohaline lakes derived from seawater are characterized by the predominance of sodium and chloride ions and a proportional composition of ions similar to that of seawater [2,50]. Besides, potassium, calcium, and magnesium are important cations, and fluoride, bromide, bicarbonate, and sulfate are important anions in thalassohaline lakes [51]. The ionic composition of Tuz Lake, containing potassium, calcium, magnesium as important cations, with a predominance of sodium and chloride ions (Table S1) unequivocally corroborates that this lake is a thalassohaline environment. Similar physico-chemical characteristics were determined in a recent study of this lake [31].

The high salinity (≥30%) of Tuz Lake, Deep Zone, and the three salterns determined a very scarce prokaryotic diversity, showing in all of them a highly similar and restricted phyla composition, and a high dominance of the phylum *Euryarchaeota*. These data are in agreement with previous studies in Tuz Lake [29,30] and in other thalassohaline environments, such as crystallizer ponds in Santa Pola (Spain) and other salterns (Spain) [36,52,53]. Besides, *Halobacteria* was the most abundant class in our study and several other thalassohaline lakes such as Great Salt Lake [54,55,56], Lake Tyrell [13,57,58], Aran-Bidgol salt lake [59], Lake Meyghan [60], and in the ponds of Santa Pola saltern [36,37,61] and Isla Cristina saltern in Spain [61,62].

In the present study, *Haloquadratum* (24%) and *Salinibacter* (1.5%) were respectively found as the most abundant archaeal and bacterial genera in Tuz Lake, the latter in a much lower proportion with respect to haloarchaea, but being the most relevant bacterial representative on these hypersaline habitats. Our data are in agreement with a previous study based on DGGE and 16S rRNA gene libraries of brine samples collected from two locations of the lake, in which *Haloquadratum* and *Salinibacter* were detected as the main genera [29]. The presence of representatives of the genera *Halobacterium*, *Haloquadratum*, *Halorhabdus*, and *Halorubrum* was noted in another study of Tuz Lake [30]. In the present study, a greater diversity was observed (Table 2 and Figure 3).

The dominant presence of the genus *Haloquadratum* was also observed in a NaCl saturated crystallizer pond (salinity 37%) of Santa Pola saltern near Alicante (Spain) [36] and in the three salterns of Salitral Negro (SN), Colorada Grande (CG), and Guatraché (G) in Argentina [53]. Researchers stated that the genera *Halorubrum* and *Haloquadratum* were respectively found as the dominant taxa in marine salterns of Isla Cristina and Santa Pola (Spain) [61]. Furthermore, *Haloquadratum, Haloarcula, Natronomonas*, and *Halorubrum* were determined as the dominant genera in crystallizer ponds of Chula Vista salterns (salinity 32–35%), near San Diego, California (U.S.A.) [63]. High abundance of *Salinibacter* in the hypersaline crystallizer ponds of Santa Pola salterns, Alicante (Spain) at salinities ranging from 19 to 37% were also reported in previous studies [36,64,65,66]. Members of the extremely halophilic genus *Salinibacter* share the same habitats with the haloarchaeon *Haloquadratum* [3,67]. It has been emphasized that since the phages that infect *Salinibacter* were more active than the phages that infect *Haloquadratum*, the presence of *Haloquadratum* was found higher than that of *Salinibacter* in the environment [68]. The high prevalence of *Haloquadratum* in hypersaline environments may be related to the production of halomucin, which enables the growth of *Haloquadratum walsbyi* at the limits of water activity. Halomucin may protect *Haloquadratum* cells from cations and halophages found in hypersaline environments [69].

In addition to the dominance of *Haloquadratum* and a low abundance of *Salinibacter*, we also detected representatives of the genera *Haloarcula* and *Halorhabdus* in high abundance, and of the genera *Halorubrum*, *Halonotius*, *Natronomonas*, *Halolamina*, and *Halobacterium* in lower abundances in the Tuz Lake. Unlike previous studies carried out in this lake, we report for the first time the presence of two new dominant taxa belonging to uncultured *Halobacteria* and a third less abundant uncultured *Halobacteria* taxon (Table 2), contributing to the heterotrophic activity in this environment, but with unknown characteristics and functional activities in those habitats to be determined in future studies.

Currently, extremely halophilic archaea, also called haloarchaea, are taxonomically classified within *Halobacteria*, a class of the phylum *Euryarchaeota*, which are considered as the most halophilic microorganisms, growing from about 10% (*w*/*v*) NaCl to saturated salt concentration. It consists of three orders: *Halobacteriales*, *Haloferacales*, and *Natrialbales*, which include six families and more than 50 genera [5,70]. A recent pan-genome analysis and ancestral state reconstruction of the class *Halobacteria* suggest a new super-order comprising of *Natrialbales* and *Halobacteriales* [71]. Our study in Tuz Lake has shown that eleven haloarchaeal genera are part of the microbiota of this environment (Table 2 and Figure 3), and that they are very diverse with respect to their taxonomic affiliations. In fact, these 11 genera are included in two different orders: *Haloferacales* and *Halobacteriales*. Representatives of the first order are placed on genera belonging to two different families: *Haloferacaceae* (genus *Haloquadratum*) and *Halorubraceae* (genera *Halorubrum*, *Halonotius* and *Halolamina*), while the order *Halobacteriales* included representatives placed on the families *Halobacteriaceae* (genera *Halobacterium* and *Haloplanus*) and *Haloarculaceae* (genera *Haloarcula*, *Halorhabdus*, *Natronomonas*, *Halosimplex*, and *Halomicrobium*). This is a much diverse archaeal community than those observed previously in this lake and several other similar thalassohaline lakes.

In the present study, we determined a lower diversity of extremely halophilic archaea in the Deep Zone (Table 2 and Figure 3), which contained higher Na^+^ and Cl^−^ concentrations compared to the other sampling sites (Table S1). *Haloquadratum* is considered the most hyperhalophilic organism known and dominates NaCl supersaturated thalassic lakes where the other halophilic archaea are eliminated by the uninhabitable conditions [72]. Moreover, the concentration of NaCl in the crystallizer ponds due to evaporation of the saline water finally results in the precipitation of sodium chloride (halite); consequently, a MgCl_2_ rich brine is produced. Before the magnesium chloride brines get sterile, *Haloquadratum* dominates and can make up to almost 80% of the total microbial biomass [36,37,61,73]. Therefore, at extremely high salinity, some essential nutrients (e.g., phosphates) become unavailable due to complexation with Mg^2+^ [74]. The chemical composition of the water in the Deep Zone is significantly different with respect to Tuz Lake, which may affect the precipitation reactions; Ca and Mg precipitates are formed in the Deep Zone [75]. In our study, while mean values of Na^+^, Ca^2+^, and Cl^−^ concentrations of the Deep Zone were found to be higher than that of the Tuz Lake, mean values of K^+^ and Mg^2+^ concentrations were lower than that of the lake. These data support the above-mentioned conclusions of Bolhuis et al. [74] and indicate that some of the essential nutrients may be biologically unavailable in the Deep Zone, causing the selection of some extreme halophiles. Therefore, we suppose that the high Na^+^ concentrations and the deficiency of some essential nutrients in the Deep Zone support less diverse extremely halophilic archaeal populations and almost all sequences from the Deep Zone belonged to the OTUs classified as representatives of the genera *Haloquadratum* and *Halorhabdus*, and the uncultured *Halobacteria* TLH-2 (Figure 3). In addition, it is obvious that the halophilic diversity in the Deep Zone is not affected by the lake because the sill in the north-south direction has separated the Deep Zone from the main lake as the water level has decreased over the recent years.

On the other hand, another factor that could influence the haloarchaeal taxonomic composition of hypersaline habitats is the extracellular DNA metabolism by some haloarchaea. Hypersaline aquatic environments have been reported to contain high extracellular DNA concentrations and that DNA can be used primarily as a phosphorus source. DNA is utilized selectively as a phosphorus source, with a bias against highly divergent methylated DNA [76]. Recent studies by Hua et al. [77] have shown that microbial communities of Isla Cristina saltern (Spain) utilized extracellular DNA only as a phosphorus source and that the taxonomic composition of these communities changed with the availability of inorganic phosphorus or extracellular DNA, with preferential uptake of extracellular DNA from specific haloarchaeal taxa, especially from the most abundant ones. These authors concluded that some haloarchaea prefer extracellular DNA from closely related taxa and that the microbial community assembly is driven by the available resources, including carbon, extracellular DNA, and inorganic phosphorus [77].

In the present study, the genus *Haloquadratum* demonstrated a negative correlation with all haloarchaeal genera (except the genus *Halorhabdus*) and the unclassified *Halobacteria* OTU-1 (Figure 5). This is in agreement with the negative correlation of the genus *Haloquadratum* with the genera *Haloarcula*, *Halobaculum*, *Halorubrum*, *Halonotius*, and *Salinibacter* determined in Lake Tyrrell in Australia [58].

The presence and abundance of representatives of the haloarchaeal genera in Tuz Lake, its salterns, and the Deep Zone may have also been affected by halocins produced by archaeal strains in this habitat. Halocins are natural proteinaceous antimicrobials that were first discovered by Rodríguez-Valera et al. [78] and are commonly produced by haloarchaea [79]. In previous studies, we detected that the halocin production by archaeal populations in Tuz Lake and Kaldirim and Kayacik salterns were a common feature [80,81]. It has been emphasized that halocins reduce the competition with other prokaryotes by lysing competitors and enriching the environment for the producer strains [78].

## 5. Conclusions

In this study, we describe the first detailed data on the prokaryotic communities of different areas of Tuz Lake, as well as its Deep Zone and the three salterns located on the shores of the largest hypersaline lake in Turkey, by using 16S rRNA amplicon sequencing (Illumina chemistry). The high salinity (30–38%) and other physico-chemical and environmental characteristics of the sampling sites studied limit the diversity of these hypersaline habitats, with extremely halophilic archaea as the dominant population, and to a lesser extent, a few bacterial representatives, with the extremely halophilic bacterial genus *Salinibacter* as the main taxon found in all samples analyzed. Prokaryotic diversity (archaea and bacteria) of the sampling sites was determined in the summer season, with high radiation and desiccation, and despite the high extension of the area studied, a similar microbial composition was found among samples.

Besides the high abundance of representatives of the haloarchaeal genus *Haloquadratum*, determined in all sampling areas, especially in Tuz Lake (24%) and the Deep Zone (47%), and Yavsan (41%) and Kayacik (31%) salterns, other haloarchaeal genera were also determined in these extreme habitats: *Haloarcula*, *Halorhabdus*, *Halorubrum*, *Halonotius*, *Natronomonas*, *Halolamina*, *Halobacterium*, *Halosimplex*, *Halomicrobium*, and *Haloplanus*. It is noteworthy that, besides sequences related to previously described taxa, two uncultured extremely halophilic microorganisms designated as uncultured *Halobacteria* TLHs 1 and 2 were found to be very abundant, especially in Tuz Lake, Kaldirim saltern, and Deep Zone and Kayacik saltern, respectively. Their isolation in pure culture would give focus to their role and functional activities on these natural environments and their possible use in industrial processes and as bioindicators of environmental changes or pollution increases in this habitat, that despite being a protected area, it is relatively close to industrial and human-inhabited zones. Therefore, further research is required to determine the features of these uncultured taxa, the isolation of new strains using novel growth media and culture conditions, permitting the isolation of these extremely halophilic microorganisms, and elucidating their ecological role in these natural habitats, as well as to explore their biotechnological and biomedical potentials.

## Figures and Tables

**Table 1:** Prokaryotic richness and diversity estimates, based on 97% OTU clusters of Tuz Lake, Deep Zone, and Kayacik, Kaldirim, and Yavsan salterns. The diversity indexes: PD Whole Tree, Chao1, Shanon, Simpson, and Dominance were calculated.

Sample	PD Whole Tree	Chao 1	Observed OTUs	Shannon	Simpson	Dominance
**Tuz Lake**						
TL1A	39.02	2879.40	1517.00	6.22	0.94	0.06
TL1B	41.00	3388.46	1753.00	6.09	0.94	0.06
TL2A	77.94	8354.98	3799.00	7.50	0.95	0.05
TL2B	88.63	9402.42	4539.00	7.82	0.96	0.04
TL3A	56.38	5378.22	2756.00	6.31	0.90	0.10
TL3B	60.63	5983.23	2881.00	7.23	0.95	0.05
TL4A	53.08	3761.75	1936.00	6.75	0.95	0.05
TL4B	59.47	3958.28	2357.00	7.23	0.96	0.04
TL5A	64.97	6757.91	3134.00	8.11	0.98	0.02
TL5B	76.81	7325.52	3930.00	8.46	0.98	0.02
TL6A	57.09	5566.74	2674.00	5.38	0.77	0.23
TL6B	69.76	7010.20	3450.00	6.15	0.84	0.16
TL7A	51.98	4779.82	2134.00	5.73	0.89	0.11
TL7B	53.02	4555.30	2241.00	5.24	0.80	0.20
**Deep Zone**						
TL8A	24.94	1805.01	914.00	4.43	0.82	0.18
TL8B	20.61	1480.57	707.00	4.29	0.79	0.21
TL9A	23.76	1684.02	883.00	4.65	0.85	0.15
TL9B	26.09	2007.02	1008.00	4.55	0.83	0.17
**Kayacik saltern**						
KYS1A	71.63	8049.00	3488.00	6.98	0.91	0.09
KYS1B	58.10	6263.30	2768.00	6.94	0.91	0.09
KYS2A	40.67	3763.57	1812.00	5.52	0.90	0.10
KYS2B	34.26	2669.73	1453.00	5.31	0.90	0.10
**Kaldirim saltern**						
KS1A	78.75	8057.13	4026.00	8.27	0.98	0.02
KS1B	81.74	8825.45	4242.00	8.19	0.97	0.03
KS2A	78.01	8276.09	3692.00	7.24	0.94	0.06
KS2B	67.12	6241.22	2900.00	6.66	0.91	0.09
**Yavsan saltern**						
YS1A	32.29	2572.08	1321.00	5.16	0.82	0.18
YS1B	70.16	6340.94	3174.00	5.98	0.85	0.15
YS2A	104.00	10,259.51	5725.00	7.17	0.92	0.08
YS2B	85.79	8352.18	4468.00	7.28	0.92	0.08

**Table 2:** Taxonomic distribution at the genus level, with more than 0.3% sequences detected in the brine samples collected from Tuz Lake, Deep Zone, and Kayacik, Kaldirim, and Yavsan salterns.

Genus/Taxon	Phylum	Tuz Lake	Deep Zone	Kayacik Saltern	Kaldirim Saltern	Yavsan Saltern
**Uncultured *Halobacteria* TLH-1**	*Euryarchaeota*	24.61 ± 3.20	5.43 ± 0.28	10.65 ± 2.66	31.77 ± 4.86	17.93 ± 2.12
***Haloquadratum***	*Euryarchaeota*	23.99 ± 4.25	47.32 ± 1.74	31.33 ± 2.78	16.57 ± 3.18	41.30 ± 3.60
***Haloarcula***	*Euryarchaeota*	14.36 ± 4.32	0.03 ± 0.00	1.80 ± 0.79	14.52 ± 6.39	5.46 ± 1.42
**Uncultured *Halobacteria* TLH-** **2**	*Euryarchaeota*	13.98 ± 1.04	22.99 ± 1.78	36.18 ± 5.85	14.14 ± 1.51	16.40 ± 2.16
***Halorhabdus***	*Euryarchaeota*	6.90 ± 0.93	23.76 ± 1.95	14.22 ± 2.93	6.40 ± 0.69	8.82 ± 1.78
***Halorubrum***	*Euryarchaeota*	3.87 ± 1.34	0.15 ± 0.02	1.80 ± 0.42	2.07 ± 0.46	2.26 ± 0.60
***Halonotius***	*Euryarchaeota*	3.25 ± 0.66	0.02 ± 0.00	0.96 ± 0.41	4.69 ± 1.65	1.50 ± 0.51
***Natronomonas***	*Euryarchaeota*	3.00 ± 1.55	0.02 ± 0.00	0.30 ± 0.15	2.99 ± 1.39	0.81 ± 0.24
***Halolamina***	*Euryarchaeota*	1.73 ± 0.91	<0.01	0.27 ± 0.23	2.15 ± 0.17	0.39 ± 0.24
***Salinibacter***	*Rhodothermaeota*	1.54 ± 0.37	0.09 ± 0.03	0.88 ± 0.42	1.09 ± 0.16	2.10 ± 1.13
***Halobacterium***	*Euryarchaeota*	1.19 ± 0.53	0.01 ± 0.00	0.04 ± 0.02	0.44 ± 0.19	0.51 ± 0.27
***Halosimplex***	*Euryarchaeota*	0.58 ± 0.11	0.08 ± 0.01	0.37 ± 0.14	1.34 ± 0.43	0.67 ± 0.23
***Halomicrobium***	*Euryarchaeota*	0.23 ± 0.05	0.01 ± 0.00	0.17 ± 0.05	0.84 ± 0.36	0.30 ± 0.14
***Haloplanus***	*Euryarchaeota*	0.16 ± 0.04	0.01 ± 0.00	0.46 ± 0.11	0.42 ± 0.09	0.60 ± 0.11
**Uncultured *Halobacteria* TLH-** **3**	*Euryarchaeota*	0.07 ± 0.02	0.00 ± 0.00	0.06 ± 0.03	0.22 ± 0.05	0.34 ± 0.14

## Data Availability

Sequence data are available at the European Nucleotide Archive (ENA)/NCBI, study accession number PRJNA705280.

## Supplementary Materials

The following are available online at https://www.mdpi.com/article/10.3390/microorganisms9071525/s1. Table S1: Environmental parameters and physico-chemical analyses results of 30 brine samples collected from Tuz Lake (TL1A to TL7B), Deep Zone (TL8A to TL9B), and Kayacik (KYS1A to KYS2B), Kaldirim (KS1A to KS2B), and Yavsan (YS1A to YS2B) salterns. Table S2: Mean percentages of phyla composition of brine samples collected from Tuz Lake, Deep Zone, and Kayacik, Kaldirim, and Yavsan salterns. Table S3: Principal component analysis of physico-chemical parameters. Figure S1: Rarefaction curves plotting the number of observed OTUs as a function of the number of sequences.
