# Supplementary material for: Prokaryotic Communities in the Thalassohaline Tuz Lake, Deep Zone, and Kayacik, Kaldirim and Yavsan Salterns (Turkey) Assessed by 16S rRNA Amplicon Sequencing
Source: Microorganisms. 2021 Jul 17;9(7):1525. doi: 10.3390/microorganisms9071525 (PMC8304926; doi:10.3390/microorganisms9071525)
Supplement: Supplementary file 1 [file microorganisms-09-01525-s001.zip › microorganisms-1286516-supplementary.pdf]

Table S1. Environmental parameters and physico-chemical data of the 30 brine samples collected from Tuz Lake (TL1A to TL7B), Deep Zone (TL8A to TL9B), and Kayacik (KYS1A to KYS2B), Kaldirim (KS1A to KS2B) and Yavsan (YS1A to YS2B) salterns.

Sample	Latitude	Longitude	Temperature (°C)	pH	Salinity (%)	Na^{+} (mg L^{-1})	K^{+} (mg L^{-1})	Mg^{2+} (mg L^{-1})	Ca^{2+} (mg L^{-1})	Cl^{-} (mg L^{-1})
**Tuz Lake**										
TL1A	39° 00' 40.0" N	33° 24' 53.2" E	25	7.2	34	74000	11025	22859	186	179932
TL1B	39° 00' 40.0" N	33° 24' 53.2" E	25	7.2	34	75521	10468	25296	174	182573
TL2A	39° 00' 35.2" N	33° 23' 28.1" E	25	7.1	34	79063	7755	19381	155	196109
TL2B	39° 00' 35.2" N	33° 23' 28.1" E	25	7.1	34	79500	10391	21950	199	197430
TL3A	38° 50' 03.3" N	33° 24' 57.8" E	27	7.3	32	92663	9694	9728	230	209315
TL3B	38° 50' 03.3" N	33° 24' 57.8" E	27	7.3	32	96333	6763	7981	295	214928
TL4A	38° 48' 39.0" N	33° 26' 31.4" E	27	7.3	32	91354	8568	19559	197	203042
TL4B	38° 48' 39.0" N	33° 26' 31.4" E	27	7.3	32	85800	7945	19863	180	199080
TL5A	38° 47' 04.3" N	33° 12' 16.3" E	25	7.0	36	74154	11520	19699	158	181583
TL5B	38° 47' 04.3" N	33° 12' 16.3" E	25	7.0	36	74594	8344	20373	140	180922
TL6A	38° 46' 23.1" N	33° 13' 07.5" E	25	7.2	34	85316	7980	17351	213	199741
TL6B	38° 46' 23.1" N	33° 13' 07.5" E	25	7.2	34	82957	8339	25332	201	198090
TL7A	38° 57' 22.3" N	33° 24' 48.9" E	22	6.9	38	62449	3767	31221	134	173329
TL7B	38° 57' 22.3" N	33° 24' 48.9" E	22	6.9	38	63089	10373	31424	130	174649
**Deep Zone**										
TL8A	38° 48' 27.6" N	33° 35' 40.7" E	24	7.4	30	91878	4976	8995	299	203372
TL8B	38° 48' 27.6" N	33° 35' 40.7" E	24	7.4	30	94129	13869	8161	285	210636
TL9A	38° 45' 54.1" N	33° 37' 33.5" E	24	7.4	30	122228	5391	8384	322	203042
TL9B	38° 45' 54.1" N	33° 37' 33.5" E	24	7.4	30	94000	5181	8340	281	209645
**Kayacik saltern**										
KYS1A	38° 50' 03.3" N	33° 25' 01.4" E	27	7.3	32	90000	4821	12139	273	202052
KYS1B	38° 50' 03.3" N	33° 25' 01.4" E	27	7.3	32	93688	6540	11486	325	209645
KYS2A	38° 48' 39.0" N	33° 26' 32.8" E	27	7.4	30	95875	4357	8246	462	211956
KYS2B	38° 48' 39.0" N	33° 26' 32.8" E	27	7.4	30	91419	3118	6652	444	203703
**Kaldirim saltern**										
KS1A	39° 00' 37.5" N	33° 24' 49.9" E	25	7.3	32	77865	10757	26212	188	196439
KS1B	39° 00' 37.5" N	33° 24' 49.9" E	25	7.3	32	80319	3227	23115	160	199741
KS2A	39° 00' 36.3" N	33° 23' 29.9" E	25	6.9	36	64649	11950	34404	141	204033
KS2B	39° 00' 36.3" N	33° 23' 29.9" E	25	6.9	36	59980	9395	29610	128	206344
**Yavsan saltern**										
YS1A	38° 47' 01.7" N	33° 12' 16.5" E	25	7.4	32	91974	3636	8333	416	204693
YS1B	38° 47' 01.7" N	33° 12' 16.5" E	25	7.4	32	88311	4153	8370	491	202382
YS2A	38° 46' 26.6" N	33° 13' 04.0" E	25	7.3	32	90405	5891	12756	335	203042
YS2B	38° 46' 26.6" N	33° 13' 04.0" E	25	7.3	32	88813	4188	10000	375	203372

Table S2. Mean percentages of phyla composition of brine samples collected from Tuz Lake, Deep Zone, and Kayacik, Kaldirim and Yavsan salterns.

Sampling Region	*Euryarchaeota*	*Parvarchaeota*	*Actinobacteria*	*Rhodothermaeota*	*Cyanobacteria*	*Firmicutes*	*Lentisphaerae*	*Proteobacteria*	*Spirochaetes*	Unassigned	Other
**Tuz Lake**	98.09 ± 0.37	< 0.01	< 0.01	1.54 ± 0.37	< 0.01	0.04 ± 0.02	0.02 ± 0.01	0.10 ± 0.07	< 0.01	0.18 ± 0.04	0.03 ± 0.02
**Deep Zone**	99.84 ± 0.05	-	-	0.09 ± 0.03	-	< 0.01	-	-	-	0.07 ± 0.03	0.01 ± 0.00
**Kayacik Saltern**	98.74 ± 0.51	< 0.01	< 0.01	0.89 ± 0.42	< 0.01	0.01 ± 0.00	< 0.01	0.01 ± 0.00	-	0.36 ± 0.17	< 0.01
**Kaldirim Saltern**	98.72 ± 0.15	-	< 0.01	1.11 ± 0.16	< 0.01	0.01 ± 0.00	0.01 ± 0.01	0.02 ± 0.01	-	0.14 ± 0.03	0.01 ± 0.01
**Yavsan Saltern**	97.49 ± 1.28	-	< 0.01	2.13 ± 1.16	-	0.03 ± 0.02	< 0.01	0.08 ± 0.07	< 0.01	0.26 ± 0.07	0.01 ± 0.01

Table S3. Principal component analysis of physico-chemical parameters.

Physico-chemical parameters	Principal components	
	**Dim1**	**Dim2**
**Temperature (°C)**	0.236	0.600
**pH**	0.409	0.047
**Salinity (%)**	-0.407	-0.083
**Sodium (mg L^{-1})**	0.388	-0.017
**Potassium (mg L^{-1})**	-0.232	0.648
**Magnesium (mg L^{-1})**	-0.399	0.111
**Calcium (mg L^{-1})**	0.364	-0.294
**Chloride (mg L^{-1})**	0.340	0.335

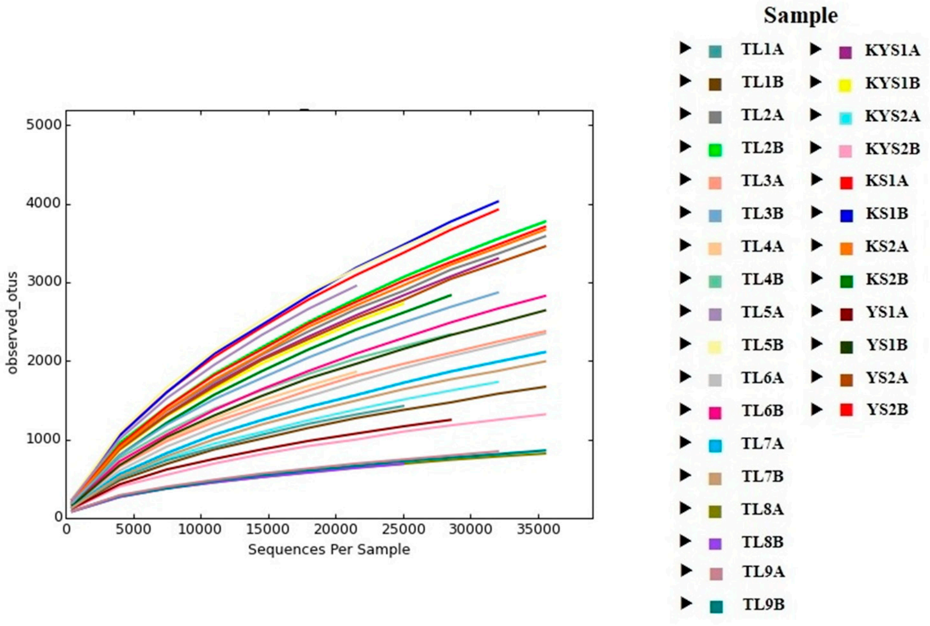

Figure S1. Rarefaction curves plotting the number of observed OTUs as a function of the number of sequences.
